# Long COVID symptoms and duration in SARS-CoV-2 positive children — a nationwide cohort study

**DOI:** 10.1007/s00431-021-04345-z

**Published:** 2022-01-09

**Authors:** Luise Borch, Mette Holm, Maria Knudsen, Svend Ellermann-Eriksen, Soeren Hagstroem

**Affiliations:** 1 Department of Pediatric and Adolescent Medicine, NIDO Denmark, Hospitalsenheden Vest, Gl. Landevej 61, 7400 Herning, Denmark; 2 Pediatrics and Adolescent Medicine, Aarhus University Hospital, Aarhus, Denmark; 3 Unit of Clinical Biostatistics, Aalborg University Hospital, Aalborg, Denmark; 4 Department of Clinical Microbiology, Aarhus University Hospital, Aarhus, Denmark; 5 Steno Diabetes Center North Denmark, Aalborg University Hospital, Aalborg, Denmark; 6 Department of Pediatrics, Aalborg University Hospital, Aalborg, Denmark

**Keywords:** Sars-CoV-2, Children, COVID-19, Long COVID, Long-term recovery

## Abstract

Most children have a mild course of acute COVID-19. Only few mainly non-controlled studies with small sample size have evaluated long-term recovery from SARS-CoV-2 infection in children. The aim of this study was to evaluate symptoms and duration of ‘long COVID’ in children. A nationwide cohort study of 37,522 children aged 0–17 years with RT-PCR verified SARS-CoV-2 infection (response rate 44.9%) and a control group of 78,037 children (response rate 21.3%). An electronic questionnaire was sent to all children from March 24th until May 9th, 2021. Symptoms lasting > 4 weeks were common among both SARS-CoV-2 children and controls. However, SARS-CoV-2 children aged 6–17 years reported symptoms more frequently than the control group (percent difference 0.8%). The most reported symptoms among pre-school children were fatigue Risk Difference (RD) 0.05 (CI 0.04–0.06), loss of smell RD 0.01 (CI 0.01–0.01), loss of taste RD 0.01 (CI 0.01–0.02) and muscle weakness RD 0.01 (CI 0.00–0.01). Among school children the most significant symptoms were loss of smell RD 0.12 (CI 0.12–0.13), loss of taste RD 0.10 (CI 0.09–0.10), fatigue RD 0.05 (CI 0.05–0.06), respiratory problems RD 0.03 (CI 0.03–0.04), dizziness RD 0.02 (CI 0.02–0.03), muscle weakness RD 0.02 (CI 0.01–0.02) and chest pain RD 0.01 (CI 0.01–0.01). Children in the control group experienced significantly more concentration difficulties, headache, muscle and joint pain, cough, nausea, diarrhea and fever than SARS-CoV-2 infected. In most children ‘long COVID’ symptoms resolved within 1–5 months.

*Conclusions*: Long COVID in children is rare and mainly of short duration.

**Table:** 

**What is Known:** *• There are increasing reports on ‘long COVID’ in adults.* *• Only few studies have evaluated the long-term recovery from COVID-19 in children, and common for all studies is a small sample size (median number of children included 330), and most lack a control group.*	
**What is New:** *• 0.8% of SARS-CoV-2 positive children reported symptoms lasting >4 weeks (‘long COVID’), when compared to a control group.* *• The most common ‘long COVID’ symptoms were fatigue, loss of smell and loss of taste, dizziness, muscle weakness, chest pain and respiratory problems.* *• These ‘long COVID’ symptoms cannot be assigned to psychological sequelae of social restrictions.* *• Symptoms such as concentration difficulties, headache, muscle- and joint pain as well as nausea are not ‘long COVID’ symptoms.* *• In most cases ‘long COVID’ symptoms resolve within 1-5 months.*

**Supplementary information:**

The online version contains supplementary material available at 10.1007/s00431-021-04345-z.

## Introduction

Compared to adults, children have a milder course of acute COVID-19 [1, 2]. Moreover, SARS-CoV-2 prevalence is lower in the paediatric population. Hence, paediatric cases constitute 11.8% (age 0–17 years), 15.6% (age 0–18 years) and 10% (age 0–19 years) in USA, Italy and Denmark, respectively [3–5]. In Denmark, only 0.08% of children with COVID-19 needed hospitalization [6]. Worldwide, deaths from COVID-19 in children also remain rare, at 0.17 per 100.000 population, comprising 0.48% of the estimated total mortality from all causes in a normal year [7].

As the cumulated incidence of SARS-CoV-2 infection increases, a growing concern arises on persistent multiorgan symptoms after the acute infection, commonly known as ‘long COVID’. Long COVID is used to describe signs and symptoms that continue or develop after acute COVID-19, and not explained by an alternative diagnosis. To date, there is no clear agreement on the definition or duration for this syndrome. According to NICE guideline, ‘long COVID’ includes both on-going symptomatic COVID-19 (from 4 to 12 weeks after acute covid-19) and post-COVID-19 syndrome (12 weeks or more after acute covid-19) [8].

There are increasing reports on ‘long COVID’ in adults [2, 9–12]. However, only few studies have evaluated the long-term recovery from COVID-19 in children [13–26]. Common for all studies is a small sample size (median number of children included 330), and most lack a control group. These studies reported that 4–66% of children experienced post-acute COVID-19 symptoms including insomnia, respiratory symptoms, nasal congestion, fatigue, muscle and joint pain, concentration difficulties and loss of smell and taste [13–26]. The lack of larger paediatric studies including a control group demonstrates a need for further epidemiological data collection in order to quantify and characterize ‘long COVID’ in children and adolescents.

The aim of this study was to document symptoms and duration of ‘long COVID’ in a nationwide cohort of SARS-CoV-2 infected children < 18 years. A control group of children who had not been tested positive for SARS-CoV-2 was included for evaluation of symptom relation to SARS-CoV-2 infection.

## Materials and methods

We conducted a national cohort study of 37,522 children aged 0–17 years with verified SARS-CoV-2 infection by polymerase chain reaction (RT-PCR) and a control group of 78,037 randomly selected children, who had not been tested positive for SARS-CoV-2. None of the children included had received a COVID vaccination. The study was approved by The Danish Health Data Authority and registered at the Central Denmark region (# 1–16-02–621-20). Ethical approval was not required according to Danish law.

At birth or time of immigration, all Danish citizens are assigned a unique personal identification number registered in the Danish Civil Population Register [27]. This is used in all healthcare contacts, facilitating confidential linkage between registers in Denmark. For the present study, The Danish Health Data Authority linked social security numbers from all Danish children to the national microbiology database. This linkage generated a complete list of all Danish children aged 0–17 years with RT-PCR verified SARS-CoV-2 infection at any date between January 27th, 2020, and March 19th, 2021.

An electronic questionnaire (REDCap) was sent to all SARS-CoV-2 positive children as well as to the control group from March 24th until May 9th, 2021. The questionnaire was sent out twice during this period, including a reminder after 7–10 days. Both groups of children had a 4-week response time.

The SARS-CoV-2 positive group had the questionnaire sent directly to the parents’ secure and private digital post-box (e-boks), which is linked to social security numbers. Adolescents from 15 years received the questionnaire themselves due to legal rights, with advice to fill it out with parental support. The questionnaire was sent out to 6.674 children aged 0–5 years, and 30.848 aged 6–17 years.

For the control group, the questionnaire was sent out to parents of children aged 0–17 years, who attended public school or day-care in five municipalities in Denmark (Aalborg, Herning, Aarhus, Randers and Frederiksberg). The parents received the questionnaire directly by the school or day-care’s online communication platform. A total of 45,240 school children (age 6–17 years) and 32,797 children in day-care (age 0–5 years) received the questionnaire. In order to exclude children who had been tested positive for SARS-CoV-2 from the control group, previous SARS-CoV-2 infection was addressed in the first question with termination of the rest of the questionnaire if confirmed.

The questionnaire for both groups consisted of identical questions regarding demographic information, a history of chronic illness, medication and symptoms lasting for more than four weeks (S1). For children 9 years and older, the WHO-5 well-being index questions were included [28].

In order to compare the SARS-CoV-2-group to the control group, the children were divided into two age groups: pre-school 0–5 years and school children 6–17 years, and risk differences were estimated (RD). The comparison between SARS-CoV-2 positive children and children in the control group was made in order to distinguish symptoms of ‘long COVID’ from symptoms attributable to the pandemic such as school lockdown and social distancing. The well-being of children older than 9 years was evaluated by WHO-5 well-being index questions in order to analyse whether symptoms could reflect a low sense of well-being, and as such not a direct effect of SARS-CoV-2 infection.

Follow-up time from verified SARS-CoV-2 to time of questionnaire completion differed from child to child. The duration of reported ongoing symptoms was addressed by calculation of difference between the known date of verified SARS-CoV-2 infection and the questionnaire response date.

## Statistical analysis

Statistical analysis was done using StataMP 17.

Binomial regressions were fitted to estimate risk difference (RD) in percentage point with 95% confidence interval for each long covid symptom as a function of SARS-CoV-2.

Unpaired two-sample *t* test with unequal variances was used to determine if there was a significant difference between the means of two groups (*α* = 0.05). Pearson Chi-square test was used to test whether two categorical variables were independent.

## Results

Of the 37,522 SARS-CoV-2 infected children, a total of 16,836 (44.9%) responses were returned (44.6% age 0–5 years and 39.1% age 6–17 years). In the control group, a total of 16,620 (21.3%) responded to the questionnaire (21% age 0–5 years and 18.3% age 6–17 years).

Mean age for children 0–5 years was 2.7 years (SARS-CoV-2 positive) and 2.8 years (control group), mean difference − 0.1 (CI − 0.17 to − 0.02). Mean age for children 6–17 years was 12.0 years (SARS-CoV-2 positive) and 10.5 years (control group), mean difference 1.5 (CI 1.41–1.59).

**Fig. 1:**
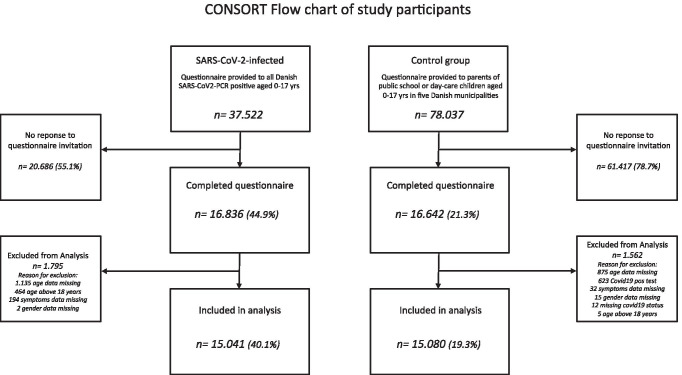
CONSORT Flow chart of study participants

Questionnaires were excluded from analyses in case of missing data regarding symptoms, age or gender of the child. When answering the questionnaire, some of the adolescents had turned 18 years since the date of their SARS-CoV-2 positive PCR test; these were excluded from the analysis. Moreover, respondents with less than 4 weeks from PCR testing were also excluded from the analysis of ‘long COVID’. A total of 15.041 SARS-CoV-2 positive children and 15.080 children in the control group were eligible for inclusion in the study (Fig. 1).

**Fig. 2:**
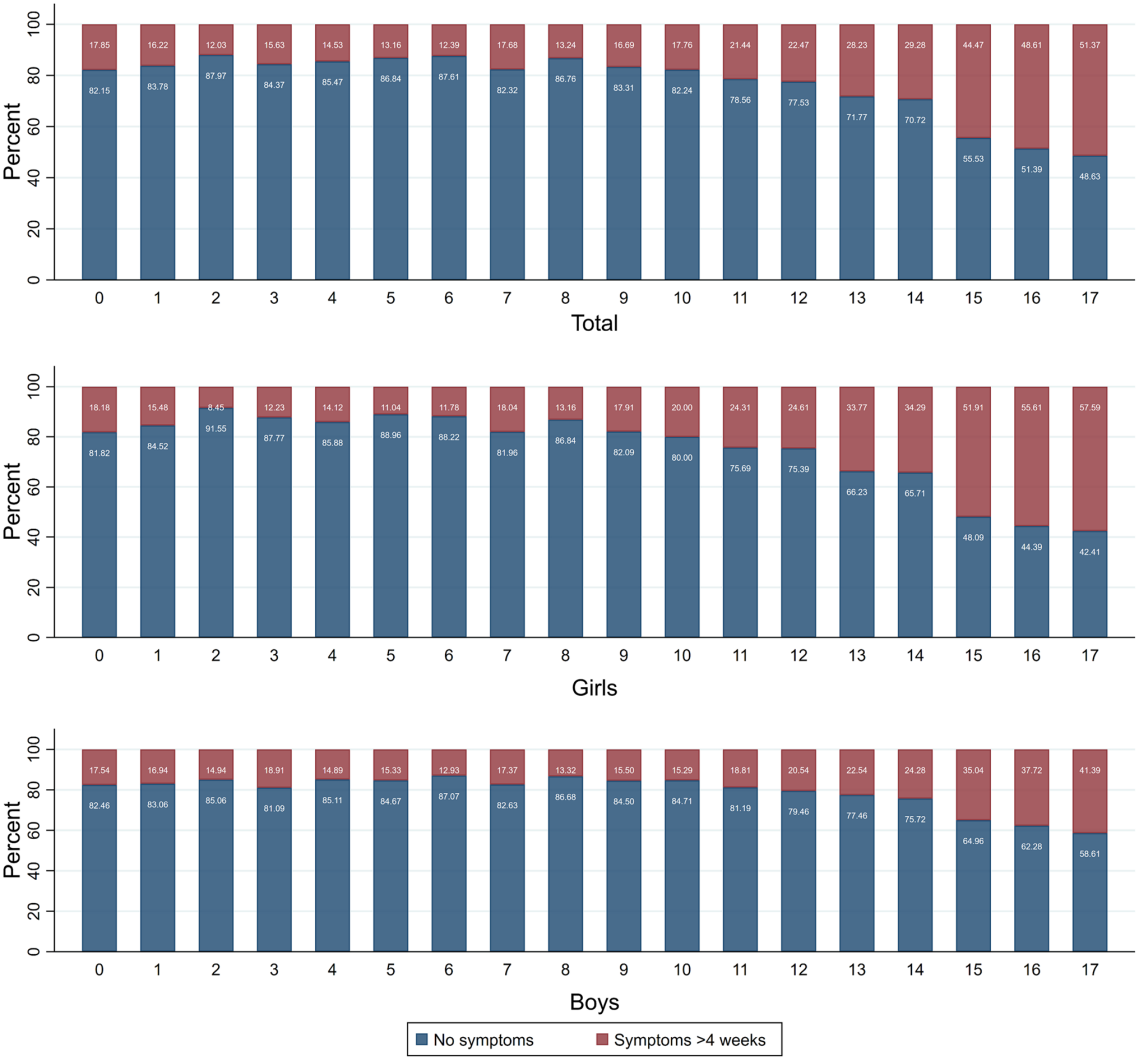
Prevalence of symptoms lasting > 4 weeks. Percentage of SARS-CoV-2 infected children reporting at least one symptom lasting > 4 weeks (red bars) or reporting no symptoms (blue bars). Data are presented as total population of SARS-CoV-2 infected children (upper panel) and by gender (girls, middle panel; boys, lower panel)

**Fig. 3:**
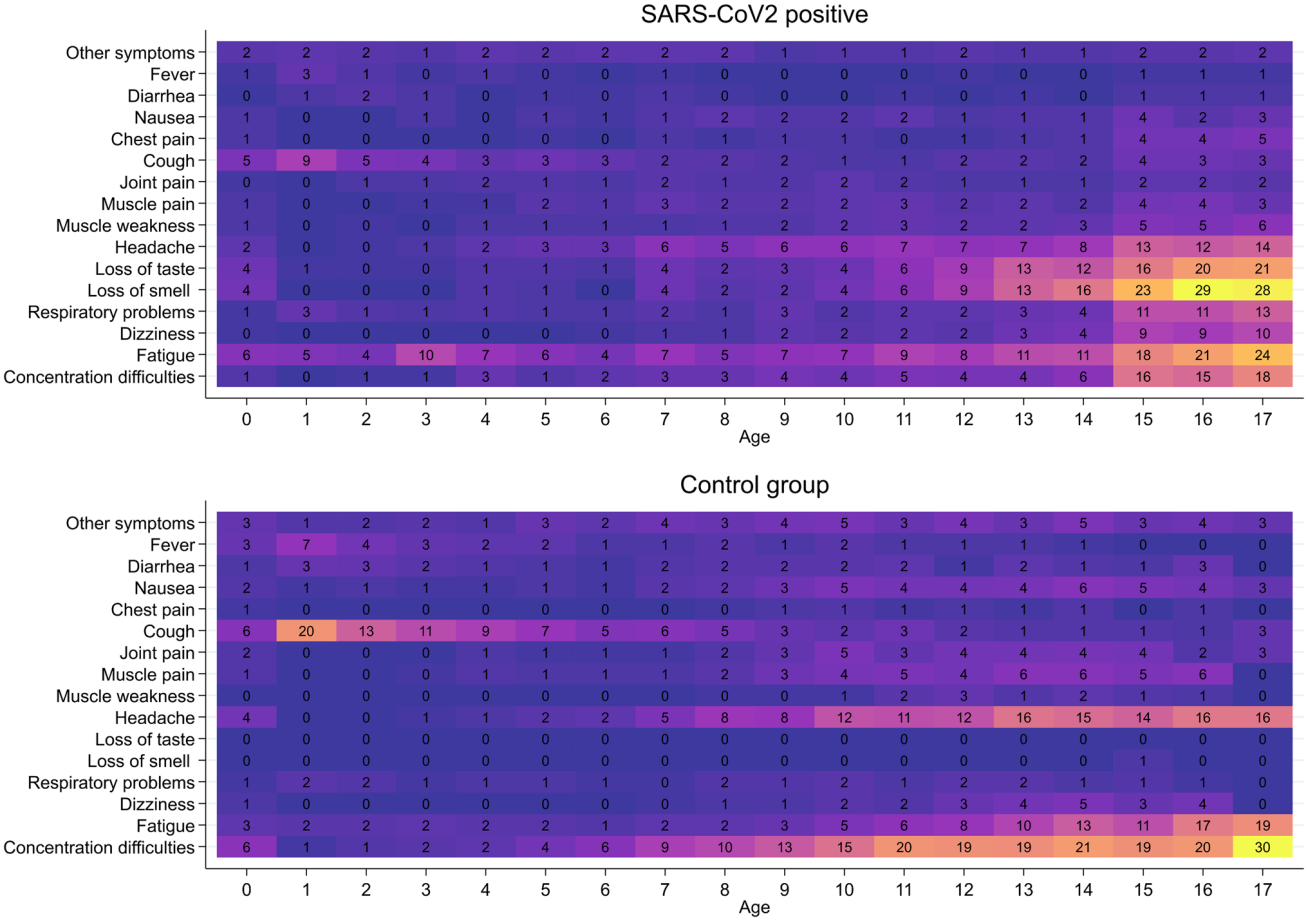
Heatmap illustrating reported symptoms lasting for > 4 weeks by SARS-CoV-2 infected children (upper panel) and controls (lower panel). The numbers represent percentage of children reporting the given symptom by one-year age groups

Depending on age, 12–51% of verified SARS-CoV-2 infected children who responded to the questionnaire experienced symptoms > 4 weeks after being diagnosed with SARS-CoV-2 infection (Fig. 2). The number of SARS-CoV-2 infected children who experienced symptoms increased with increasing age (Fig. 2), pre-school children 439 of 2.976 (14.8%) vs school children 3.374 of 12.065 (28%); *p* = 0.000. The most common symptoms were fatigue, loss of smell and loss of taste, headache and concentration difficulties (Fig. 3).

Of the children in the control group responding to the questionnaire, 15–38% (depending on age) experienced symptoms lasting > 4 weeks, pre-school children 1.201 of 6.832 (17.6%) vs school children 2.245 of 8.248 (27.2%); *p* = 0.000. In the control group, the most commonly reported symptoms were concentration difficulties, cough, headache and fatigue (Fig. 3).

Five percent of children in both groups suffered from a chronic disease, mainly a respiratory diagnosis (51% of the SARS-CoV-2 children with a chronic disease reported a respiratory diagnosis).

Within the age group 0–5 years, more children in the control group reported symptoms lasting > 4 weeks compared to SARS-CoV-2 positive children (14.8% vs 17.6%; *p* = 0.001, difference − 2.8%). Within the age group 6–17 years, 0.8% more SARS-CoV-2 positive children reported symptoms lasting > 4 weeks than children in the control group (28% vs 27.2%; *p* = 0.020, difference 0.8%).

The burden of symptoms was higher among SARS-CoV-2 positive children compared to children in the control group (*p* < 0.0001). Given that, of the 3813 SARS-CoV-2 positive children who reported symptoms lasting > 4 weeks, 1323 children (34.7%) reported one symptom, 1095 children (28.7%) reported two symptoms and 1395 children (36.6%) reported three or more symptoms. Of the 3446 children in the control group who reported symptoms lasting > 4 weeks, 1870 children (54.3%) reported one symptom, 794 children (23%) reported two symptoms and 782 children (22.7%) reported three or more symptoms.

**Fig. 4:**
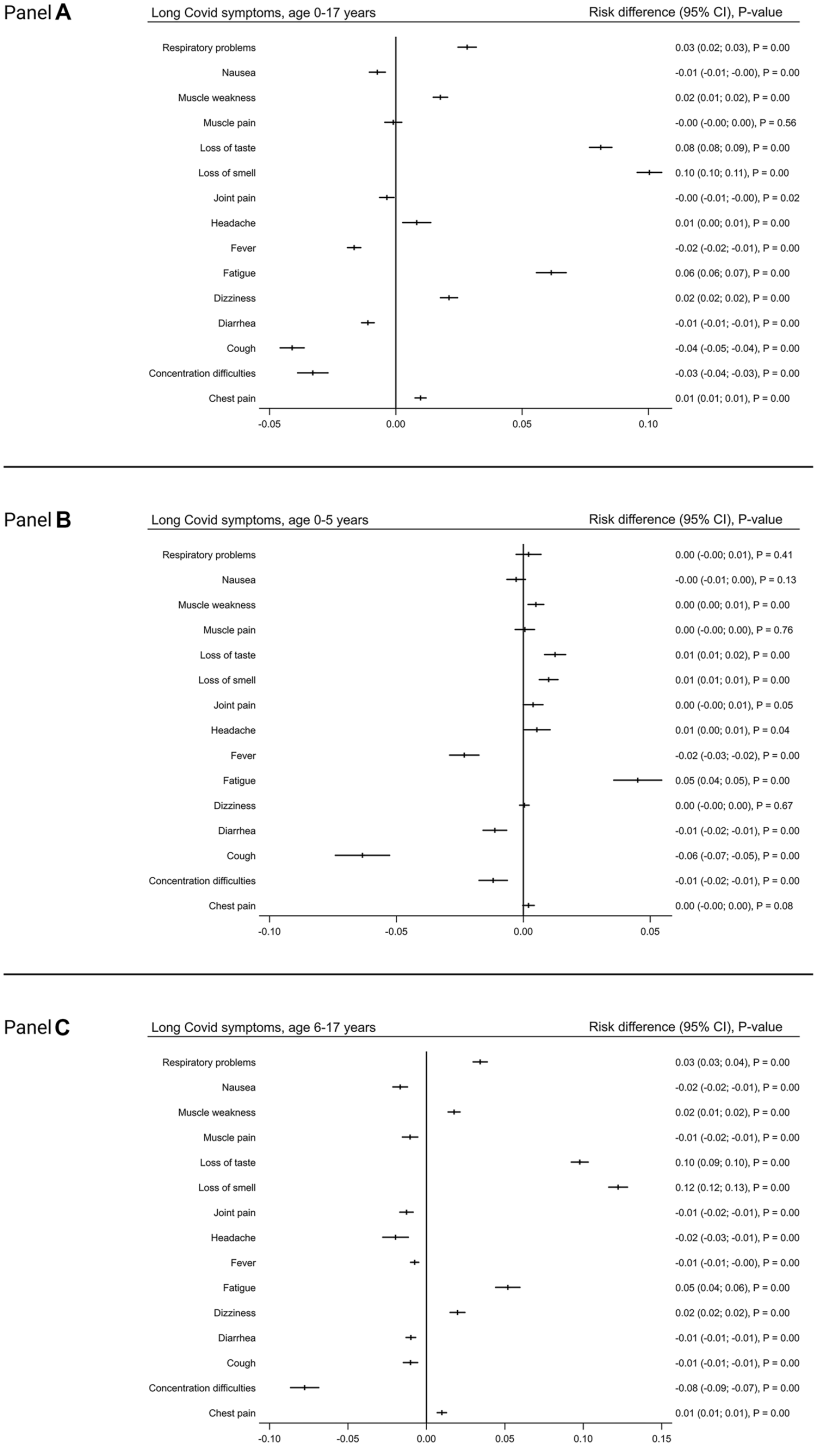
Comparison of symptom prevalence in SARS-CoV-2 infected children and the control group indicated by risk differences (RD) with 95% confidence interval and *p*-values. Panel **A**: comparison of children 0–17 years. Panel **B**: Sub-group comparison of pre-school children, 0–5 years. Panel **C**: Sub-group comparison of school children, 6–17 years

Some symptoms were more frequent among SARS-CoV-2 children while other symptoms were more frequent among children in the control group. SARS-CoV-2 pre-school children more often suffered from fatigue RD 0.05 (CI 0.04–0.06), loss of smell RD 0.01 (CI 0.01–0.01), loss of taste RD 0.01 (CI 0.01–0.02) and muscle weakness RD 0.01 (CI 0.0–0.01) (Figs. 4 and S2). Correspondently, SARS-CoV-2 school children more often suffered from loss of smell RD 0.12(CI 0.12–0.13), loss of taste RD 0.10 (CI 0.09–0.10) and fatigue RD 0.05 (CI 0.05–0.06). The risk difference was less but still significant in reported respiratory problems RD 0.03 (CI 0.03–0.04), dizziness RD 0.02 (CI 0.02–0.03), muscle weakness RD 0.02 (CI 0.01–0.02) and chest pain RD 0.01 (CI 0.01–0.01) (Figs. 4 and S2).

Children in the control group age 0–5 years experienced significantly more cough, fever, concentration difficulties and diarrhoea than children in the SARS-CoV-2 group. Correspondently, 6–17-year-old controls were more prone to concentration difficulties, headache, nausea, muscle and joint pain, cough, diarrhea and fever than their SARS-CoV-2 positive peers (Figs. 4 and S2).

Seven percent of the SARS-CoV-2 infected children, who reported symptoms lasting > 4 weeks, were asymptomatic during the acute infection.

**Fig. 5:**
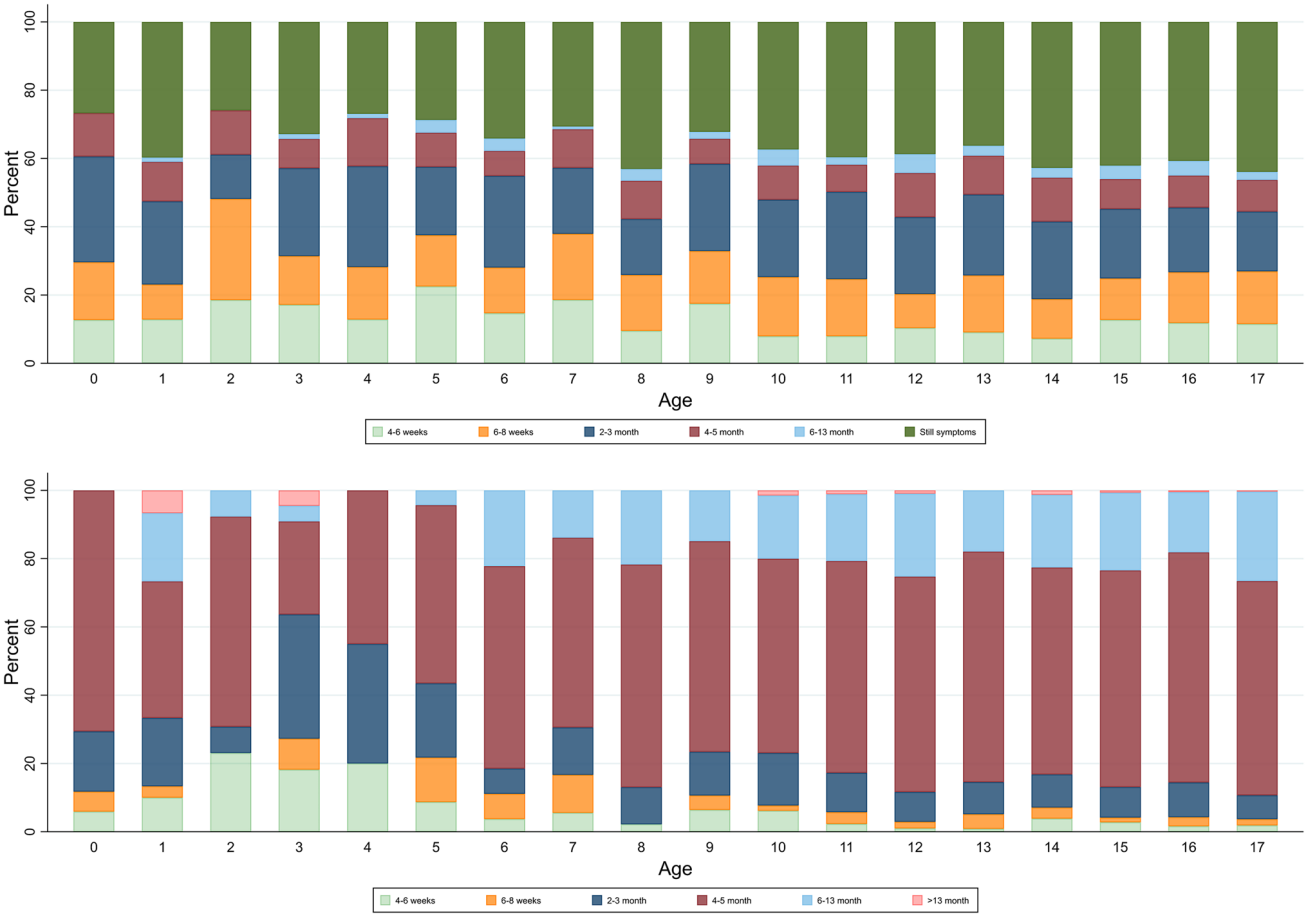
Duration of symptoms from date of positive RT-PCR SARS-CoV-2 test until date for questionnaire completion by 1-year age groups. In the upper panel, the dark green bars represent percentage of children who reported that their symptoms had not resolved at time of questionnaire completion. The follow-up time of these children´s on-ongoing symptoms is illustrated in the lower panel

Depending on age, symptoms resolved in a minimum of 54–75% of children within 1–5 months (Fig. 5, panel A). Thirty-nine percent of SARS-CoV-2 infected respondents reported ongoing symptoms at the date of answering the questionnaire. Length of follow-up for the children reporting on-going symptoms is illustrated in Fig. 5, panel B.

Comparison of the questionnaire responders in the control group to the SARS-CoV-2 infected children documented that SARS-CoV-2 infected had a higher sense of well-being compared to the control group, WHO-score difference 4 (CI 3.5–4.8). Children in both groups, who experienced symptoms for > 4 weeks, had a worse sense of well-being compared to children within the same group but without symptoms, SARS-CoV-2 positive: WHO-score difference 12 (CI 11.1–12.8) control group: WHO-score difference 17.2 (CI 16.2–18.3).

## Discussion

To our knowledge, this is the largest study documenting symptoms and duration of ‘long COVID’ in a national cohort comparing RT-PCR-verified SARS-COV-2 infected children to a control group of randomly selected children, who have not been tested positive for SARS-CoV-2.

During the COVID-19 pandemic, governmental interventions have been introduced in order to reduce transmission of SARS-CoV-2. Quarantine regimes, increased hand hygiene, school and day-care lock down, closure of sports and leisure activities and social distancing are among the numerous interventions. Reports have raised concern on the negative impact of these social implications on children’s mental health [29]. Bearing this in mind, it is crucial to compare reported ‘long COVID’ symptoms in previously SARS-CoV-2 positive children to a cohort representative of the background population. Describing ‘long COVID’ in a paediatric population without a control group could otherwise overestimate ‘long COVID’ symptoms.

In the present study, children included in the SARS-CoV-2 group and the control group did not differ in age, gender or pre-existing chronic diseases. SARS-CoV-2 positive children as well as children in the control group reported a high prevalence of symptoms lasting > 4 weeks. These symptoms could potentially originate from SARS-CoV-2 infection, non-SARS-CoV-2 infections or be symptoms reflecting psychological and social consequences of the pandemic. Unfortunately, the present study lacks data on other contemporary viral infections in the children included. However, we would expect an equal distribution of non-SARS-CoV- 2 viral infections in the two groups. Our analysis showed that SARS-CoV-2 positive children presenting with symptoms lasting > 4 weeks reported a higher sense of well-being compared to children with symptoms > 4 weeks who have never been tested positive for SARS-CoV-2. As such, the prevalence of symptoms in the SARS-CoV-2 positive group cannot be assigned to psychological sequelae of social restrictions. At the same time, it can be speculated that the observed lower WHO-5 score and high prevalence of symptoms in the control group could reflect the implications of social restrictions and psychological consequences of the pandemic.

Despite a high prevalence of reported symptoms lasting > 4 weeks in the 6–17-year-old children in the SARS-CoV-2 positive group (28.0%), only a residual percentage difference of 0.8% is seen after subtraction of the corresponding symptom prevalence in the control group (27.2%). It may be speculated that the 0.8% could be a more reliable estimate of the true ‘long COVID’ prevalence in these SARS-CoV-2 positive children. However, it should be kept in mind that this mathematical calculation does not take into accountability difference in type of symptoms reported as well as the burden of symptoms between the two groups. Previous paediatric studies found that 8–58% of SARS-CoV-2 positive children experience ‘long COVID’ symptoms [13, 15–17, 21–26]. However, none of these studies included a control group, resulting in a risk of overestimating the prevalence of ‘long COVID’. Three studies included a control group and documented a much lower prevalence of ‘long COVID’ symptoms [19, 20, 30]. Symptoms lasting > 4 weeks in controls vs cases were reported to be 1.7% vs 4.6% [30], 0.9% vs 4.4% [19] and 53.4% vs 66.5% [20], respectively. These low prevalences of ‘long COVID’ are comparable to our findings.

In accordance with previous studies [19, 23, 26], the present study documented that the age distribution of symptoms differed with older school children being more frequently affected compared to younger school and pre-school children.

Comparing the responses from SARS-CoV-2 infected children to the control group, we found that the most common statistically significant ‘long COVID’ symptoms were fatigue, loss of smell and loss of taste and to a lesser extend muscle weakness, chest pain, dizziness and respiratory problems. These symptoms are also commonly reported as ‘long COVID’ symptoms in other studies [31]. Especially fatigue and loss of smell and taste [32] have been reported as frequent symptoms among SARS-CoV-2 positive children. Fatigue has been reported in up to 85% of SARS-CoV-2 positive children with an illness duration of > 4 weeks [14–16, 19, 20, 22]. In comparison, median duration of fatigue after Epstein-Barr virus infection is 15.5 days [33].

Concentration difficulties, headache, muscle and joint pain, cough, nausea, diarrhoea and fever have previously been described as ‘long COVID’ symptoms of SARS-CoV-2 infection in children in non-controlled trials [24, 34]. However, we found that these symptoms were statistically more significant in the control group. Our study also documented that children in the control group had a lower WHO-5score compared to SARS-CoV-2 positive children. Therefore, it should be considered whether concentration difficulties, headache, muscle and joint pain and nausea could be symptoms reflecting the negative impact of the social implications of the pandemic on children’s mental and physical health. The reason for reports of more frequent fever occurrence in a randomly selected control group can only be speculated, since questions on specific method of temperature evaluation or accompanying symptoms were not included. In general, the first year of the pandemic has had a remarkably low incidence of otherwise high incidence infections, such as respiratory syncytial virus and influenza virus [35, 36]. We would therefore have expected a low occurrence of fever.

The duration of ‘long COVID’ symptoms is an important issue to address with implications for the children and families. In our paediatric cohort, most children recovered within a maximum of 1–5 months. Recovery time of 2 weeks–3 months has previously been described in paediatric studies [15, 19, 24].

There are significant limitations to this observational study. Firstly, our questionnaire was not validated in a larger pilot study. Moreover, data relies on participants’ retrospectively parent- or self-reported symptoms with a risk of recall bias. However, SARS-CoV-2 is an infection with increased focus in society. As such, participants have possibly been extremely aware of symptoms during their infection, and therefore, recall bias is assumed to be minimal. However, it can also be speculated that children in the control group, as well as the SARS-CoV-2 group who experienced symptoms, were more eager to respond to the questionnaire than those having mild symptoms or being asymptomatic. This could lead to selection bias and result in an overestimation of reported symptoms. The study may also suffer from non-response bias where non-symptomatic controls may be under-represented. The questionnaire was distributed and had to be answered online, which is likely to select participants with a high socio-economic background, who have a lower risk of poor outcomes following disease.

Secondly, the control group might include children who have had SARS-CoV-2 infection without having undergone testing. This is a risk since children have no or only few symptoms of acute COVID-19. It has previously been reported that 25–36% of SARS-CoV-2 positive children are asymptomatic [24, 37]. Moreover, parents might be more reluctant to let children undergo testing due to the invasive and unpleasant procedure. Children in the control group have never been tested positive for SARS-CoV-2. Nevertheless, seroprevalence data suggest an infection rate of 2–3 times higher than corresponding PCR results in the paediatric population [38]. In March 2021, a Danish nationwide seroprevalence surveillance study of 530 randomly selected children 12–18 years old documented that 8.1% had positive SARS-CoV-2 IgG (unpublished national data). It could be assumed that children below 12 years also account for an 8.1% seroprevalence. The potential presence of children with previous SARS-CoV-2 infection in the control group would underestimate the symptoms of ‘long COVID’.

Thirdly, the number of children answering the questionnaire was higher among SARS-CoV-2 positive children than in the control group (44.9% versus 21.3%). The higher response rate in the group of children with previous SARS-CoV-2 infection could be expected since these children and families might have higher awareness on potential ‘long COVID’ symptoms, and a potential increased desire for knowledge about post-acute sequelae of SARS-CoV-2. This issue might result in an overestimation of ‘long COVID’. However, in both groups, the children who answered the questionnaire were an equal age distribution of the children who received the questionnaire.

The strengths of this study are primarily the large sample size and inclusion of a control group. Additionally, the Danish Health Data Authority has a nationwide coverage, and all Danish citizens have universal tax-funded health insurance. Therefore, our study population did not rely on access to health-care services, and RT-PCR tests for SARS-CoV-2 are free of charge in Denmark minimizing selection bias. The infection status of all SARS-CoV-2 positive children has been established by nationally validated RT-PCR, eliminating major misclassification of infection status. Moreover, the results of the SARS-CoV-2 test are linked to the unique personal identification number registered in the Danish Civil Population Register [27] and the national microbiology database making it a complete cohort of SARS-CoV-2 infected children below 18 years from the start of the pandemic in Denmark until 19^th^ of March 2021.

The control group is not nationwide but consists of randomly selected children aged 0–17 years from five municipalities that include both larger cities as well as rural areas spread widely across Denmark. As such, the control group is considered to represent the general Danish population of children. The strengths mentioned above increase the generalizability of our results. However, it is important to consider that an observational study based on a questionnaire with subjective responses and without objective examination of the children can only provide us with one piece of the puzzle. Further studies are needed to increase the knowledge of ‘long COVID’ in the paediatric population.

In conclusion, to date, this study is the largest study on symptoms and duration of ‘long COVID’ in SARS-CoV-2 positive children also including a control group. It provides new evidence of ‘long COVID’ in children, documenting that ‘long COVID’ is primarily seen in older school children. Despite the high prevalence (12–51%) of reported long-lasting symptoms in the SARS-CoV-2 children, the true prevalence of ‘long COVID’ seems a lot lower, maybe as low as 0.8%. The most common ‘long COVID’ symptoms are fatigue, loss of smell and loss of taste, dizziness, muscle weakness, chest pain and respiratory problems. These symptoms cannot be assigned to psychological sequelae of social restrictions. Symptoms such as concentration difficulties, headache, muscle and joint pain and nausea may be related to other factors than SARS-CoV-2 infection. In most cases, ‘long COVID’ symptoms resolve within 1–5 months.

## Supplementary information

Below is the link to the electronic supplementary material.

Supplementary file1 (PDF 127 KB)

## Declarations

### Transparency statement

The lead author affirms that this manuscript is an honest, accurate and transparent account of the study being reported. No important aspects of the study have been omitted. The study has been performed as originally planned.
