# Supplementary material for: Long COVID symptoms and duration in SARS-CoV-2 positive children — a nationwide cohort study
Source: Eur J Pediatr. 2022 Jan 9;181(4):1597–607. doi: 10.1007/s00431-021-04345-z (PMC8742700; doi:10.1007/s00431-021-04345-z)
Supplement: Supplementary file 1 — Supplementary file1 (PDF 127 KB) [file 431_2021_4345_MOESM1_ESM.pdf]

Supplementary S1

Questionnaire for SARS-CoV-2 children

1. Date for completion of the questionnaire?
2. Your child's birth date including year?
3. Gender of your child?
4. Has your child ever been tested positive for SARS-CoV-2 (corona virus)?
5. If "yes" in question 4: What date did your child have a positive corona test for the first time?
6. Does you child suffer from a chronic disease?
7. If "yes" in question 6: What chronic disease?
   - Respiratory disease such as asthma, cystic fibrosis, bronchitis
   - Immune deficiency (confirmed by doctor)
   - Cancer
   - Diabetes
   - Kidney disease
   - Abdominal disease
   - Heart disease
   - Neurological disease such as epilepsy
   - Other chronic disease
8. Does your child receive any daily medication?
9. If "yes" in question 8: What type and dose of medication?
10. Did your child have any of the following symptoms during the period from the date of his/her positive covid test until 2 weeks after the test was positive?
    - Fever
    - A common cold
    - Sore throat
    - Cough
    - Respiratory problems
    - Loss of smell
    - Loss of taste
    - Headache
    - Vomiting
    - Diarrhea
    - Muscle pain
    - Joint pain
    - No symptoms
11. Is it more than 4 weeks since your child tested positive for corona virus for the first time?
12. If "yes" in question 11: Did you child have any of the following symptoms 4 weeks or more after his/her positive corona test?
    - Concentration difficulties
    - Fatigue
    - Dizziness
    - Loss of smell
    - Loss of taste
    - Headache
    - Muscle weakness
    - Muscle pain
    - Joint pain
    - Cough
    - Chest pain
    - Nausea
    - Diarrhea
    - Fever
    - Other symptoms
    - My child did not have any symptoms 4 weeks or more after his/her positive corona test

13. How long did these symptoms last?

1-2 weeks
2-4 weeks
1-2 months
3-4 months
5-6 months
7-8 months
9-10 months
11-12 months
My child still has symptoms

14. Is your child 9 years or older?

15. If "yes" in question 14: Has your child been happy and in a good mood the last two weeks?

All the time
Most of the time
A little more than half of the time
A little less than half of the time
A bit of the time
At no point in time

16. If "yes" in question 14: Has your child felt calm and relaxed the last two weeks?

All the time
Most of the time
A little more than half of the time
A little less than half of the time
A bit of the time
At no point in time

17. If "yes" in question 14: Has your child been full of energy the last two weeks?

All the time
Most of the time
A little more than half of the time
A little less than half of the time
A bit of the time
At no point in time

18. If "yes" in question 14: Has your child awakened feeling well rested the last two weeks?

All the time
Most of the time
A little more than half of the time
A little less than half of the time
A bit of the time
At no point in time

19. If "yes" in question 14: Has your child´s every day life been full of things that interest the child the last two weeks?

All the time
Most of the time
A little more than half of the time
A little less than half of the time
A bit of the time
At no point in time

Questionnaire for children in the control group

1. Date for completion of the questionnaire?
2. Your child's birth date including year?
3. Gender of your child?
4. Has your child ever been tested positive for SARS-CoV-2 (corona virus)?

If "yes" in question 4 the questionnaire was terminated without further questions.

5. Does you child suffer from a chronic disease?
6. If "yes" in question 5: What chronic disease?
    - Respiratory disease such as asthma, cystic fibrosis, bronchitis
    - Immune deficiency (confirmed by doctor)
    - Cancer
    - Diabetes
    - Kidney disease
    - Abdominal disease
    - Heart disease
    - Neurological disease such as epilepsy
    - Other chronic disease
7. Does your child receive any daily medication?
8. If "yes" in question 8: What type and dose of medication?
9. Within the last year, did your child have any of the following symptoms that lasted 4 weeks or longer?
    - Concentration difficulties
    - Fatigue
    - Dizziness
    - Loss of smell
    - Loss of taste
    - Headache
    - Muscle weakness
    - Muscle pain
    - Joint pain
    - Cough
    - Chest pain
    - Nausea
    - Diarrhea
    - Fever
    - Other symptoms
    - My child did not have any symptoms
10. How long did these symptoms last in total from the day they started until the day the symptoms ended?
    - 1-2 weeks
    - 2-4 weeks
    - 1-2 months
    - 3-4 months
    - 5-6 months
    - 7-8 months
    - 9-10 months
    - 11-12 months
11. Is your child 9 years or older?
12. If "yes" in question 11: Has your child been happy and in a good mood the last two weeks?
    - All the time
    - Most of the time
    - A little more than half of the time
    - A little less than half of the time
    - A bit of the time
    - At no point in time
13. If "yes" in question 11: Has your child felt calm and relaxed the last two weeks?
    - All the time
    - Most of the time

A little more than half of the time
A little less than half of the time
A bit of the time
At no point in time

14. If “yes” in question 11: Has your child been full of energy the last two weeks?

All the time
Most of the time
A little more than half of the time
A little less than half of the time
A bit of the time
At no point in time

15. If “yes” in question 11: Has your child awakened feeling well rested the last two weeks?

All the time
Most of the time
A little more than half of the time
A little less than half of the time
A bit of the time
At no point in time

16. If “yes” in question 11: Has your child´s every day life been full of things that interest the child the last two weeks?

All the time
Most of the time
A little more than half of the time
A little less than half of the time
A bit of the time
At no point in time

Supplementary to figure 4, S2

Long Covid symptoms 0-17 years

Reported symptom	SARS-CoV-2 positive	Controls	Total	P-value
n (%)	14883 (49.4)	15234 (50.6)	30117 (100.0)	
Concentration difficulties (Checked), n (%)	910 (6.1)	1408 (9.2)	2318 (7.7)	0.00
Fatigue (Checked), n (%)	1578 (10.6)	654 (4.3)	2232 (7.4)	0.00
Dizziness (Checked), n (%)	504 (3.4)	188 (1.2)	692 (2.3)	0.00
Respiratory problems (Checked), n (%)	611 (4.1)	187 (1.2)	798 (2.6)	0.00
Loss of smell (Checked), n (%)	1529 (10.3)	19 (0.1)	1548 (5.1)	0.00
Loss of taste (Checked), n (%)	1236 (8.3)	16 (0.1)	1252 (4.2)	0.00
Headache (Checked), n (%)	1033 (6.9)	911 (6.0)	1944 (6.5)	0.00
Muscle weakness (Checked), n (%)	373 (2.5)	108 (0.7)	481 (1.6)	0.00
Muscle pain (Checked), n (%)	330 (2.2)	346 (2.3)	676 (2.2)	0.75
Joint pain (Checked), n (%)	220 (1.5)	274 (1.8)	494 (1.6)	0.03
Cough (Checked), n (%)	397 (2.7)	1017 (6.7)	1414 (4.7)	0.00
Chest pain (Checked), n (%)	215 (1.4)	68 (0.4)	283 (0.9)	0.00
Nausea (Checked), n (%)	243 (1.6)	354 (2.3)	597 (2.0)	0.00
Diarrhea (Checked), n (%)	100 (0.7)	267 (1.8)	367 (1.2)	0.00
Fever (Checked), n (%)	84 (0.6)	333 (2.2)	417 (1.4)	0.00

		Risk difference	Lower 95% CI	Upper 95% CI	P value
Concentration difficulties	(Yes)	-0.03	-0.04	-0.03	0.00
Fatigue	(Yes)	0.06	0.06	0.07	0.00
Dizziness	(Yes)	0.02	0.02	0.02	0.00
Respiratory problems	(Yes)	0.03	0.03	0.03	0.00
Loss of smell	(Yes)	0.10	0.10	0.11	0.00
Loss of taste	(Yes)	0.08	0.08	0.09	0.00
Headache	(Yes)	0.01	0.00	0.02	0.00
Muscle weakness	(Yes)	0.02	0.02	0.02	0.00
Muscle pain	(Yes)	-0.00	-0.00	0.00	0.75
Joint pain	(Yes)	-0.00	-0.01	-0.00	0.03
Cough	(Yes)	-0.04	-0.04	-0.04	0.00
Chest pain	(Yes)	0.01	0.01	0.01	0.00
Nausea	(Yes)	-0.01	-0.01	-0.00	0.00
Diarrhea	(Yes)	-0.01	-0.01	-0.01	0.00
Fever	(Yes)	-0.02	-0.02	-0.01	0.00

Long Covid symptoms 0-5 years

Reported symptom	SARS-CoV-2 positive	Controls	Total	P-value
n (%)	2950 (30.1)	6856 (69.9)	9806 (100.0)	
Concentration difficulties (Checked), n (%)	40 (1.4)	173 (2.5)	213 (2.2)	0.00
Fatigue (Checked), n (%)	193 (6.5)	135 (2.0)	328 (3.3)	0.00
Dizziness (Checked), n (%)	6 (0.2)	11 (0.2)	17 (0.2)	0.64
Respiratory problems (Checked), n (%)	41 (1.4)	80 (1.2)	121 (1.2)	0.36
Loss of smell (Checked), n (%)	31 (1.1)	3 (0.0)	34 (0.3)	0.00
Loss of taste (Checked), n (%)	39 (1.3)	4 (0.1)	43 (0.4)	0.00
Headache (Checked), n (%)	49 (1.7)	76 (1.1)	125 (1.3)	0.03
Muscle weakness (Checked), n (%)	20 (0.7)	12 (0.2)	32 (0.3)	0.00
Muscle pain (Checked), n (%)	24 (0.8)	51 (0.7)	75 (0.8)	0.72
Joint pain (Checked), n (%)	28 (0.9)	38 (0.6)	66 (0.7)	0.03
Cough (Checked), n (%)	142 (4.8)	759 (11.1)	901 (9.2)	0.00
Chest pain (Checked), n (%)	10 (0.3)	9 (0.1)	19 (0.2)	0.03
Nausea (Checked), n (%)	19 (0.6)	63 (0.9)	82 (0.8)	0.17
Diarrhea (Checked), n (%)	25 (0.8)	134 (2.0)	159 (1.6)	0.00
Fever (Checked), n (%)	34 (1.2)	237 (3.5)	271 (2.8)	0.00

		Risk difference	Lower 95% CI	Upper 95% CI	P value
Concentration difficulties	(Yes)	-0.01	-0.02	-0.01	0.00
Fatigue	(Yes)	0.05	0.04	0.06	0.00
Dizziness	(Yes)	0.00	-0.00	0.00	0.65
Respiratory problems	(Yes)	0.00	-0.00	0.01	0.38
Loss of smell	(Yes)	0.01	0.01	0.01	0.00
Loss of taste	(Yes)	0.01	0.01	0.02	0.00
Headache	(Yes)	0.01	0.00	0.01	0.04
Muscle weakness	(Yes)	0.01	0.00	0.01	0.00
Muscle pain	(Yes)	0.00	-0.00	0.00	0.72
Joint pain	(Yes)	0.00	0.00	0.01	0.05
Cough	(Yes)	-0.06	-0.07	-0.05	0.00
Chest pain	(Yes)	0.00	-0.00	0.00	0.07
Nausea	(Yes)	-0.00	-0.01	0.00	0.14
Diarrhea	(Yes)	-0.01	-0.02	-0.01	0.00
Fever	(Yes)	-0.02	-0.03	-0.02	0.00

Long Covid symptoms 6-17 years

Reported symptom	SARS-CoV-2 positive	Controls	Total	P-value
n (%)	11933 (58.8)	8378 (41.2)	20311 (100.0)	
Concentration difficulties (Checked), n (%)	870 (7.3)	1235 (14.7)	2105 (10.4)	0.00
Fatigue (Checked), n (%)	1385 (11.6)	519 (6.2)	1904 (9.4)	0.00
Dizziness (Checked), n (%)	498 (4.2)	177 (2.1)	675 (3.3)	0.00
Respiratory problems (Checked), n (%)	570 (4.8)	107 (1.3)	677 (3.3)	0.00
Loss of smell (Checked), n (%)	1498 (12.6)	16 (0.2)	1514 (7.5)	0.00
Loss of taste (Checked), n (%)	1197 (10.0)	12 (0.1)	1209 (6.0)	0.00
Headache (Checked), n (%)	984 (8.2)	835 (10.0)	1819 (9.0)	0.00
Muscle weakness (Checked), n (%)	353 (3.0)	96 (1.1)	449 (2.2)	0.00
Muscle pain (Checked), n (%)	306 (2.6)	295 (3.5)	601 (3.0)	0.00
Joint pain (Checked), n (%)	192 (1.6)	236 (2.8)	428 (2.1)	0.00
Cough (Checked), n (%)	255 (2.1)	258 (3.1)	513 (2.5)	0.00
Chest pain (Checked), n (%)	205 (1.7)	59 (0.7)	264 (1.3)	0.00
Nausea (Checked), n (%)	224 (1.9)	291 (3.5)	515 (2.5)	0.00
Diarrhea (Checked), n (%)	75 (0.6)	133 (1.6)	208 (1.0)	0.00
Fever (Checked), n (%)	50 (0.4)	96 (1.1)	146 (0.7)	0.00

		Risk difference	Lower 95% CI	Upper 95% CI	P value
Concentration difficulties	(Yes)	-0.07	-0.08	-0.07	0.00
Fatigue	(Yes)	0.05	0.05	0.06	0.00
Dizziness	(Yes)	0.02	0.02	0.03	0.00
Respiratory problems	(Yes)	0.03	0.03	0.04	0.00
Loss of smell	(Yes)	0.12	0.12	0.13	0.00
Loss of taste	(Yes)	0.10	0.09	0.10	0.00
Headache	(Yes)	-0.02	-0.03	-0.01	0.00
Muscle weakness	(Yes)	0.02	0.01	0.02	0.00
Muscle pain	(Yes)	-0.01	-0.01	-0.00	0.00
Joint pain	(Yes)	-0.01	-0.02	-0.01	0.00
Cough	(Yes)	-0.01	-0.01	-0.00	0.00
Chest pain	(Yes)	0.01	0.01	0.01	0.00
Nausea	(Yes)	-0.02	-0.02	-0.01	0.00
Diarrhea	(Yes)	-0.01	-0.01	-0.01	0.00
Fever	(Yes)	-0.01	-0.01	-0.00	0.00
